# Case 6/2016 - The Patient is a 29-Year-Old Male with Spontaneous Closure of Ventricular Septal Defect in Adulthood

**DOI:** 10.5935/abc.20160148

**Published:** 2016-10

**Authors:** Edmar Atik

**Affiliations:** Clínica privada Dr. Edmar Atik, São Paulo, SP - Brazil

**Keywords:** Down Syndrome, Heart Septal Defects, Ventricular, Heart Defects, Congenital

**Clinical findings:** The routine examination of an asymptomatic 10-day-old male patient with Down syndrome revealed a holosystolic and mild (+ to ++ of intensity) heart murmur characteristic of low ventricular septal defect, auscultated along the left sternal margin with radiation to the right sternal margin, and normal heart sounds. In addition, there were mild thrusts in the left sternal margin, mild enlargement of the liver and mild dyspnea, requiring the use of diuretics and digoxin up to the age of 18 months. Because of the mild repercussion of the defect, routine clinical follow-up was maintained, yielding systematically similar findings on physical examination. The echocardiogram always showed the subaortic perimembranous defect, ranging from 3 to 5 mm. The electrocardiogram (ECG) was within the normal range, but the chest radiography showed mild cardiac area enlargement. Good clinical course persisted with normal life, including regular physical activity, until the last recent routine medical reassessment, which no longer revealed the heart murmur. The heart murmur might have disappeared in the time interval between the assessment at the age of 18 years, when the heart murmur was still auscultated, and the last medical assessment at the age of 29 years. The patient works in the filing sector of a private company, where he is well accepted by his peers.

**Physical examination:** good general condition, eupneic, acyanotic, normal pulses. Weight: 54 kg, height: 146 cm, right upper limb blood pressure: 100/70 mmHg, heart rate: 60 bpm. The aorta was not palpated in the suprasternal notch.

The inspection of the precordium showed neither palpable *ictus cordis* nor systolic pulsations. The heart sounds were normal and no heart murmur was auscultated. The liver was not palpable and the lungs were clean.

## Complementary tests:

**Figure 1:**
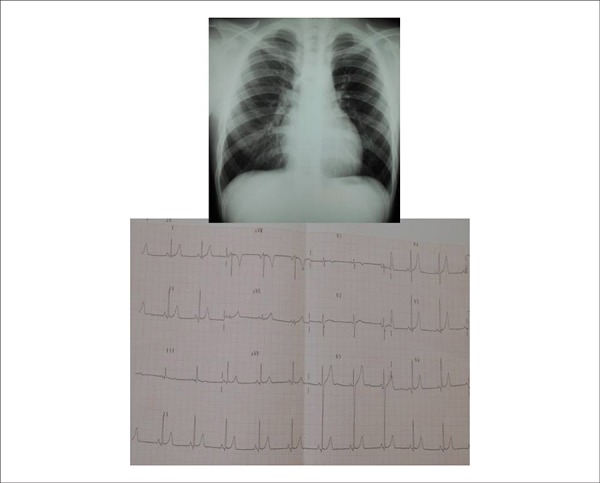
Posteroanterior chest radiography showing normal heart area and pulmonary vascular bed, and normal electrocardiographic findings at the age of 13 years, prior to closure of the ventricular septal defect.

**Electrocardiogram:** Showed sinus rhythm, no chamber overload and no changes in ventricular repolarization. AP= +40º, AQRS= +60º, AT= +30º (Figure 1). The ECGs performed before the closure of the defect were normal, in accordance with the corresponding ages.

**Chest radiography:** Showed heart area within the normal range (cardiothoracic index = 0.50) at the age of 13 years, prior to closure of the defect (Figure 1).

**Figure 2:**
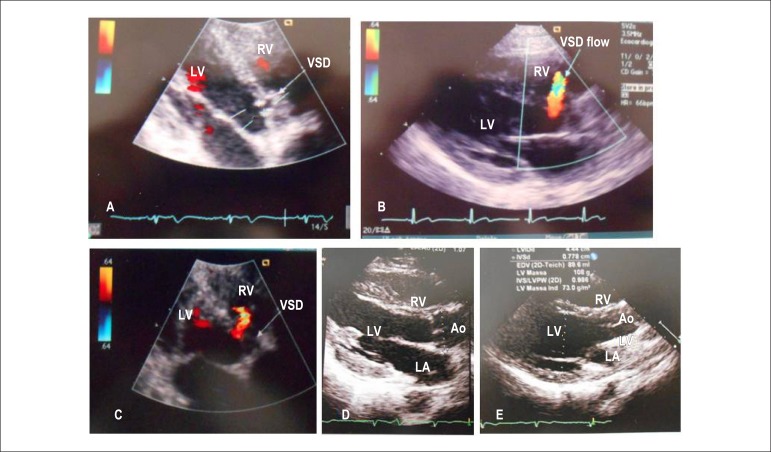
Echocardiograms showing a small subaortic ventricular septal defect at the age of 13 years in several views (A: subcostal; B: long axis; and C: apical), and no septal discontinuity in the long axis (D and E) in the recent assessment. RV: right ventricle; LV: left ventricle; LA: left atrium; Ao: aorta; VSD: ventricular septal defect.

**Echocardiogram:** Revealed heart chambers of normal size, normal biventricular function and no valvar abnormalities before and after spontaneous closure of the defect, which was located in the perimembranous region and had fibrous borders (Figure 2).

**Clinical diagnosis:** Small ventricular septal defect of mild repercussion, with spontaneous closure in adulthood and normalization of the clinical parameters.

**Clinical rationale:** The clinical findings during follow-up were compatible with the diagnosis of cardiovascular normality. Disappearance of the systolic heart murmur characteristic of ventricular septal defect previously auscultated indicated spontaneous and clear closure of the defect, which occurred in the time interval between the last assessment at the age of 18 years and the current assessment at the age of 29 years. In addition, the normal size of the heart area on chest radiography emphasized the anatomical and functional normality.

**Differential diagnosis:** The same progression can be observed in congenital heart diseases with spontaneous closure of defects, such as atrial septal defect, patent ductus arteriosus and regression of mild pulmonary valve stenosis, in addition to heart diseases surgically repaired with subsequent anatomical and functional normalization, such as ventricular and atrial septal defects, patent ductus arteriosus, aortic coarctation, transposition of great arteries and anomalous pulmonary venous drainage.

**Management:** Because of the anatomical and functional normalization, a healthy and normal life was recommended, with no restriction of any human activity.

**Comments:** The anatomical and functional normalization after the correction of the above mentioned heart defects occurs very often as long as the patients are operated upon at an early age, and neither residual defects nor residual acquired phenomena persist. The same can be seen in some defects that close spontaneously, such as atrial and ventricular septal defects, patent ductus arteriosus and mild pulmonary valve stenosis with natural involution. Most patients, specifically those with mild ventricular septal defect, experience spontaneous closure of the defect during their first year of life (75%), but the condition can extend up to the age of 5 years (23%) or even longer, up to adulthood, although rarely.^1^ That closure occurs in mild defects, whose diameters measure less than 3 to 4 mm, and rarely in larger ones.^1,2^
